# Can You Do That Again? Time Series Consolidation as a Robust Method of Tailoring Gesture Recognition to Individual Users

**DOI:** 10.3390/s22197512

**Published:** 2022-10-03

**Authors:** Louis J. Dankovich, Monifa Vaughn-Cooke, Sarah Bergbreiter

**Affiliations:** 1 A. James Clark School of Engineering, University of Maryland, College Park, MD 20742, USA; 2 Department of Mechanical Engineering, Carnegie Mellon University, Pittsburgh, PA 15213, USA

**Keywords:** gesture recognition, machine learning, classification, wearable sensors, inter-set testing

## Abstract

Robust inter-session modeling of gestures is still an open learning challenge. A sleeve equipped with capacitive strap sensors was used to capture two gesture data sets from a convenience sample of eight subjects. Two pipelines were explored. In FILT a novel two-stage algorithm was introduced which uses an unsupervised learning algorithm to find samples representing gesture transitions and discards them prior to training and validating conventional models. In TSC a confusion matrix was used to automatically consolidate commonly confused class labels, resulting in a set of gestures tailored to an individual subject’s abilities. The inter-session testing accuracy using the Time Series Consolidation (TSC) method increased from a baseline inter-session average of 42.47 ± 3.83% to 93.02% ± 4.97% while retaining an average of 5.29 ± 0.46 out of the 11 possible gesture categories. These pipelines used classic machine learning algorithms which require relatively small amounts of data and computational power compared to deep learning solutions. These methods may also offer more flexibility in interface design for users suffering from handicaps limiting their manual dexterity or ability to reliably make gestures, and be possible to implement on edge devices with low computational power.

## 1. Introduction

The hands are the most widely used body part for gesturing aside from the face and represent one of the most intuitive methods of enabling human-machine interaction [1,2,3]. Hand gesture recognition is a key element in a wide variety of domains including VR, assistive devices for disabled users, prosthetics, physical therapy devices, and computer peripherals both for general use and disabled users [2,3,4,5]. Hand gestures are classified as either static or dynamic gestures [3,6]. A static gesture is simply a pose or position of the hand that is stationary, and a dynamic gesture is composed of a series of sequential static gestures [3,6]. While there is a growing interest in the use of dynamic gestures tracked across multiple frame inputs for uses such as sign language, single frame inputs are the building block that make up dynamic gestures.

Three major types of sensor systems used in gesture recognition include data gloves, electromyography, and video based gesture recognition [3,4,5,6]. Data gloves may integrate magnetic systems, flex sensors, or inertial motion unit data [5]. They have high accuracy in static gesture recognition and can provide rich data on position, but can be cumbersome and uncomfortable to wear over an extended period of time [2]. Electromyography (EMG) leaves the hands free but suffers from signal drift for a number of reasons including sweat, fatigue, variation of muscle force, and shifting electrodes [3,6,7]. While video based solutions leave the body unencumbered by wearables their challenges include changes in lighting, issues segmenting the hands from background clutter, and occluded lines of sight [3,6].

In most studies reviewed, an application was used which cued gestures and saved sensor data along with the ground truth label of the requested gesture [8,9,10,11,12,13]. This is an attractive option for real world use given that home users are unlikely to have alternate sensors to provide ground truth labelling; but the assumption that subjects accurately follow the on-screen prompts is not always accurate. Typically, there is some period of time given to transition between gestures, which is flagged with a ‘Null’ label. This allows for three primary sources of error in labelling (i) users do not instantaneously transition between gestures and as a result many of the ‘Null’ samples will be members of actual gesture classes, (ii) user fatigue can lead to variation in production of gestures confounding a trained model [7,8,14,15], (iii) users may inadvertently make incorrect gestures [8,16]. The solutions presented in this paper focus on the first two issues.

The ‘Null’ label is typically assigned to the samples recorded during transitions between labeled gestures, and may also be assigned to hand positions that do not represent a known gesture class. Up to 75% of the samples in a given data set can be ‘Null’ gestures which do not a gesture category included in the model [17]. The majority of contemporary studies reviewed for this paper indicated they discarded transient data in beginning/in between/at end of gestures, or indicated that transitions between gestures received some manual processing [8,9,10,11,12,15]. This can lead to unrealistically optimistic performance estimates.

It is commonly observed that gesture production is subject to variability over time [8,14,15]. If a user finds a gesture difficult or uncomfortable to make, their production of the gesture may degrade over time or they may assume different wrist positions to ease gesture production [14,15]. These physical adaptions can lead to some gesture classes strongly resembling each other over multiple repetitions as the user behavior does not match the assigned label. Within image recognition, the question of how to learn from poorly labelled data has been explored using iterative training models and deep learning [18]. These solutions generally deep learning models, and as indicated by Kim et al. this may require more training data than a user will have the patience to provide at the beginning of a use session [19].

Another often unaddressed challenge is producing robust inter-set results. Often only a single data set is used to train, test, and validate models [5]. While this can produce high accuracies, it often results in significant overtraining and models that fail to generalize to new data [12]. It has been observed in a variety of studies that the accuracy of inter-set validation even on the same day tends to be far lower than in-set validation [8,10,11,12]. This in part can be attributed to signal drift, sensors shifting on users’ arms, fatigue, or even variations in user position leading to changes in sensor readings [3,4,7,9,10,12]. These changes violate a fundamental assumption in machine learning that data is stationary over time, i.e., the mean and standard deviation can be known a priori from a sample and will not change over time [10]. The field of concept drift focuses on addressing cases where the underlying data features’ relationship with the variable they are predicting changes over time. Most proposed solutions for concept drift require either an assumption that data is Gaussian in distribution (which may be incorrect in real world gesture classification), a priori knowledge of classes so that error can be detected, and fresh labelled data [20,21]. Many of the proposed solutions are dependent on deep learning. While deep learning can achieve high accuracy and recognize large numbers of gestures; it requires a significant quantity of data and high computational power [4,7]. This may represent a challenge both for edge computing devices and user patience.

While a typical goal in studies is to maximize the number of recognizable gestures encoded by a sensor, Jiang et al. note in their 2021 review on gesture recognition ‘it is unrealistic and unnecessary to capture and classify every possible hand and finger pose, and instead defining a target hand gesture set can enable adequate performance for a given specific application’ [3]. If only a small number of gestures are needed, it may be reasonable to train on a larger number of gestures and tailor the set to the ones likely to be robustly recognized for a given user. This could represent a simpler solution which could be accomplished with a classical learning algorithm. Classical algorithms do not typically recognize as many gestures, but they require far lower demands in terms of quantity of data and computational power [2,3].

In this study we examine possible solutions to both problems described in this section. For the ‘Null’ label error issue we use an unsupervised learner to find gesture transitions without manual intervention, and use a rules-based approach to repair labels on samples incorrectly placed into ‘Null’. To address the issue of inaccurate production of gestures, we leverage an averaged Confusion Matrix (CM) created from a time series train/test split using multiple repetitions of each gesture to find gestures confused with each other on a by user basis. We then consolidate gestures available to tailor them to the individual subject. In both inter-session validation is used to avoid overly optimistic performance assessments.

### 1.1. Similar and Related Works

Relatively few studies have investigated the problem of identifying modeled gestures within a set of samples containing ‘Null’ data and transitions between gestures. In 2015, Rossi et al. explored the problem of transitions between gestures in a six-gesture set using EMG sensors. They observed that over 66% of the errors in their study occurred during gesture transitions and proposed a two stage model using Hidden Markov Models (HMM) as a first state classifier to identify samples representing transitions between gestures prior to classification using Support Vector Classification (SVC) and increased their accuracy on streamed gesture data by 12% achieving a final accuracy of 84% on six gestures including the transition or ‘Null’ class [13]. A study by Taranta et al. in 2022 used computer vision to pick dynamic gestures out of streams of data which included gestures that did not fit any of their models trained classes. They used algorithms to assign confidence that a gesture belonged in a given class, and reject classifications which fell below the assigned confidence and achieved a 93.3% overall accuracy in picking 10 dynamic gestures out of a stream of data. They reported that the thresholds of confidence had to be tailored to a given user and gesture [22].

A greater number of authors have approached the problem of inter-session accuracy in recent years though many dropped or hand edited the transitional gestures which may have led to overly optimistic assessments of the end results [8,10,11,12]. Palermo et al. collected data from 10 subjects using EMG sensors and repeating 7 gestures 12 times, twice a day for 5 days. They indicated that data relabeling was performed offline to correct for subjects making incorrect gestures and found that on inter-session tests accuracy on average decreased by 27.03% [8]. Leins et al. performed an 11-gesture study using Electrical Impedance Tomagraphy on five subjects and found losses of accuracy as high as 27% for inter-session tests. The study indicates that no data was recorded for transitions between gestures. A small portion of data from the new session was used to recalibrate models and inter-session accuracies as low as 19.55% were improved to 56.34% [12]. Asfour et al. collected pressure sensor data from nine subjects performing sixteen gestures in three sessions; one session was used for training and the others for validation. All of their sessions were recorded on the same day without removing sensors, there was a ‘brief break between sessions’, and gesture transitions/‘Null’ gesture samples were discarded. They implemented a feature engineering approach in which Fischers’ Discriminant Analysis was used to transform features to a vector space maximizing the variance between classes and minimizing the variance within classes to optimize models for inter-session validation resulting in an average inter-session accuracy of 82% [11]. In their 2021 paper Moin et al. conducted a real time experiment with two participants using a 66 sensor EMG array to classify 13 gestures via an HD computing algorithm [10]. In-session their algorithm results achieved a 97.12% accuracy on 13 gestures. Inter-session tests resulted in an average 11.89% loss of accuracy; injecting fresh data from the new session could bring their intersession accuracy as high as 94.71%. However, they indicated that transitional periods between gestures required a 500 ms delay to rise to accuracies higher than 80% [10]. The only study that did not require fresh data to recalibrate was Asfour et al., and only Moin et al. included transitions between gestures.

### 1.2. Focus of This Work

In this pilot study we explore inter-session (IS) trial results utilizing a capacitive strap based sensor from our previous work on wearable gesture recognition [23]. We collect two sets of labeled data consisting of 10 gesture classes and a ‘Null’ label for transitions between gestures (‘Null’) from a convenience sample of eight subjects. We explore two different methods of processing gesture data collected from a user in order to correct for the limitations of computer assigned ground truth.

The first method which we refer to as FILT is a two-stage algorithm. An unsupervised learner finds the points in ‘Null’ containing the actual transitions between gestures under the assumption that they are outliers. In the context of this experiment rules are then used to relabel samples prior to the transitions into the previous class, and post transition samples into the next class. This allows for more accurate data to be used to train a model and a more reasonable assessment of the model’s accuracy, and is possible because in the supervised training setting used in this experiment the class labels before and after ‘Null’ are known. In an online use case the FILT method could be used to discard samples indicating that a transition between gestures was occurring and only pass actual gesture classes to a second classifier for identification.

The second method is referred to as Time Series Consolidation (TSC). Here we make the assumption that due to limitations of a sensor system or individual user some gesture classes will not be distinguishable from each other between use sessions. The algorithm works by training and testing on different repetitions of a gesture in-session, generating a confusion matrix (CM) for each time batch of repetitions, and averaging the CMs when all reps have been tested. It uses the averaged CM to find classes that fail to achieve a set threshold of accuracy and merge them with the class they are most commonly confused with. This produces a smaller set of gesture classes tailored to the user and more likely to remain robust in inter-session tests. TSC has the advantage of allowing rules to be assigned to control which classes are retained and provide a more intuitive user interface.

Finally, we explore combining the FILT method of correcting labels of ‘Null’ gestures via an unsupervised learner and the TSC method of merging of classes via use of a confusion matrix to arrive at a set of gestures tailored to limitations of the subject and/or sensor system being used to recognize gestures. To complement fine tuning the gesture set to the individual subject we also fine tune the feature set, feature normalization method, classification model, and hyper-parameters to the individual user.

## 2. Materials and Methods

We start with an overview of how the prototype used in this study was constructed, the experimental protocol used to collect data, how data was processed and cleaned, and then explore the results of various machine learning (ML) algorithms applied to the data to train predictive models.

### 2.1. Sensor Construction

**Figure 1:**
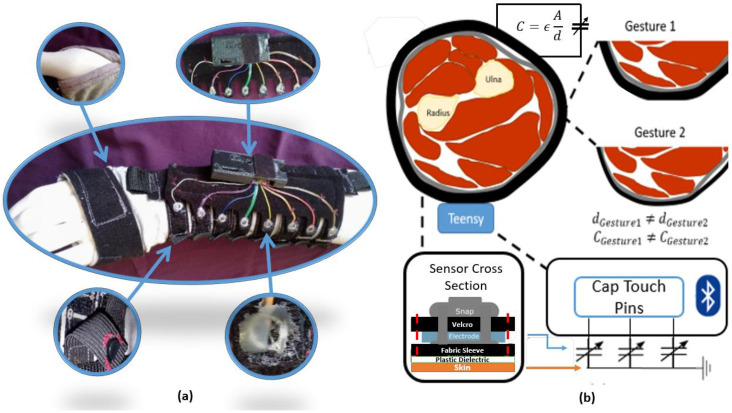
(**a**) The sleeve used in testing, each band is one of the capacitive strap sensors. The callouts from top left and moving counterclockwise are a thumb loop which helps ensure consistent position on the users’ arms, the removable electronic components, the snaps which provide connectivity between the electronics and sensors while allowing electronics to be removed, Velcro straps provide a secure attachment for the electronics themselves. To ensure consistent pressure between wear cycles image hook and eye connectors are attached to a short elastic strap as shown in highlighted portion, (**b**) A conceptual image highlighting the arm cross-section monitored by one of the 8 sensor straps on a subject’s arm. Because the area of the strap and dielectric are fixed, flexion/relaxation of the muscles leads to variation in the distance between the skin (ground) and the electrode. This results in a variation of the measured capacitance. These measurements are transmitted from the Teensy microcontroller to a computer for offline processing via Bluetooth.

We constructed an adjustable, wearable capacitive sensing sleeve for the forearm out of inexpensive, readily available materials and based on our previous work [19]. The sensors straps were composed of a conductive fabric electrode sandwiched between a 6-mil plastic sheet that acted as a waterproof dielectric and a Velcro top layer, and an embedded snap puncturing the conductive and Velcro layers was used to connect wire leads to the electronic components. A short piece of elastic was stitched to the end of the sensor strap, and equipped with hook and eye closures to allow comfort and consistent pressure when placed on subjects of a variety of sizes. These sensors were stitched over the knit fabric sleeve to ensure even spacing of sensors and a comfortable fit on subjects. This sleeve can be seen on a mannequin arm in Figure 1a. The energy stored in the electric field between the sleeve’s electrodes and the body (ground) can be measured as capacitance, governed by:

(1) $$C=\varepsilon \frac{A}{d}$$

where *ε* represents the dielectric constant of the material separating the electrodes of overlapping area *A*, separated by a distance, *d*.

The general principle of capacitive touch sensors is that they measure the change in capacitance which occurs when humans interact with a charged electrode. In these systems, the human interacting with the electrode acts as an electrical ground. In typical capacitive touch systems, the electrode is embedded in a rigid surface [24]. Instead, this approach embeds the electrode in a soft, wearable sleeve in which the electrodes wrap completely around the arm. In this circumferential electrode approach, the charged electrode is wrapped with a constant pressure around the user’s forearm. The area of the electrodes interface with the human wearer and the dielectric constant of the space between the user and the electrode remain nearly constant as the user changes gestures [23]. When the user changes gestures, the cross-sectional circumference of the muscles used in making the gesture changes due to contraction or relaxation. This in turn changes the capacitive measurements because the quantity of skin at a given distance from the electrode changes. A conceptual cartoon can be seen in Figure 1b. The capacitance of the interaction between the skin and the electrode was measured via the Teensy 3.2 microcontroller’s capacitive touch pins, which use a dual oscillator method to determine the difference in the charging and discharging times between the capacitor of interest and a reference capacitor on the microcontroller. This difference in time is reported in counts, a measure that is directly proportional to the capacitance, and then sent via an HC-05 Bluetooth module serial connection to a computer for post-processing.

### 2.2. Experiment

A convenience sample of 8 participants was recruited (4 male; 4 female; 31.9 ± 9.8 years of age; 8 right hand dominant). Exclusion criteria were individuals younger than 18 years of age and individuals with missing fingers. Prior to participation, all participants provided written informed consent prior to perform activities involved in the experiment and to publish on the results. The protocol was approved by the University of Maryland College Park Institutional Review Board (1473266-4).

**Figure 2:**
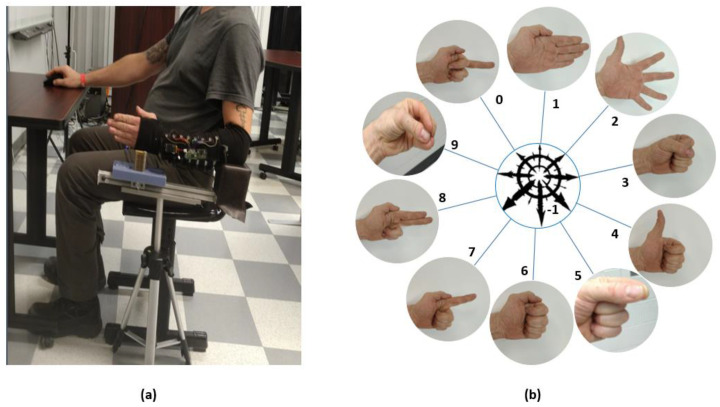
(**a**) Sensor sleeve and armrest used to ensure consistent placement of arm, (**b**) the gestures used in the study. The gestures shown are −1 Null, 0: Point middle Finger, 1: Neutral Position, 2: Spread Fingers, 3: Fist, 4: Thumb Up, 5: Pinch, 6: Thumb Adduct, 7: Point Index, 8: Point Two Finger, 9: Chuck Grasp.

A plastic wrap barrier was wrapped around subjects’ arms for hygiene and the subjects’ arm diameters were measured just above ulnar process and at the thickest part of their forearm using a standard fabric circumference tape measure (Renpho RF-BMF01). Subjects were then seated and provided with an arm rest constructed for the study to limit elbow motions and to ensure that their arm stayed in a consistent position relative to the body as seen in Figure 2a.

For the study, 10 gestures were selected cover a variety of activities of daily living and determine the limitations of the capacitive strap sensors across a variety of users. As seen in Figure 2b, the gestures selected included a variety of whole hand motions and grasps (Neutral, Spread Fingers, Pinch, Fist, Pinch, Chuck Grasp), gestures meant to cover different positions of the thumb (Thumb Adduction, Thumb Up, Fist), gestures meant to determine if individual finger positions could be registered (Point Index, Point Middle, Point Two Fingers), and a Null class label to represent the motions made while transitioning between gesture classes. A custom application was built to communicate with the electronic sensors over Bluetooth, prompt gestures, label samples, and store data for offline interpretation. To avoid potential effects of user motion including variation in limb positions, users were seated in front of a monitor which displayed the requested gesture, and an arm rest was provided to assist user in keeping arm in a fixed position with shoulder adducted and neutrally rotated, elbow flexed at 90°, forearm and wrist in neutral positions. Subjects were instructed not to make any motions except for transitioning between gestures when prompted by the application and to make gestures as naturally as possible. Five repetitions of each gesture were performed during the trial. Subjects were prompted by the application to either hold a gesture for 4 s or given a 3 s countdown to transition to the next displayed gesture. Samples were streamed from the sensors to the application over Bluetooth with a collection rate of 20 Hz. The application labeled sensor data with the prompted gesture as ground truth, or ‘Null’ for the transitional period between. Gestures were presented in a randomized order and could at times include the same gesture multiple times in a row. Each subject performed one short practice set to ensure that gestures cues from the interface were understood and then two separate datasets (Data1, Data2) were collected following the same protocol. Subjects were given a one-minute break between the data sets to rest and move around. Before and after each test, measurements of the position of the sleeve relative to the back of the armrest were taken to determine if changes in position were a possible confounding factor.

### 2.3. Machine Learning

Machine learning, data processing, and statistical analysis were performed in Python (f3.9.8) optimized for an AMD processor. The libraries used included scikit-learn (1.0.1), imblearn (0.8.1), featurewiz (0.1.7), scipy (1.8.0), scikit_posthocs (0.7.0), statsmodels (0.13.2), and bioinfokit (2.0.8). The feature sets used, scaling methods, unsupervised learning, and supervised classifiers were tailored to each user using an f1 value obtained by training and validating models on the Data1 as defined in the metrics Section 2.3.6. During preliminary trials, it was found that a 35/65 train/test split was effective in circumventing the bias-variance tradeoff and preventing models from overtraining on the Data1 dataset. The pipelines used, scaling methods, unsupervised/supervised learning models, and algorithm are described in the following subsections.

#### 2.3.1. Pipelines

For purposes of this paper, the machine learning pipeline consists of: a feature set as discussed in Section 2.3.2; scaling method as discussed in Section 2.3.2; unsupervised learning model as discussed in Section 2.3.3; and a supervised classification model as discussed in Section 2.3.4. For the scaling method, “None” was provided as an available option because it was hypothesized that variations of user position and unrequested motions could invalidate the assumptions of scaling models as discussed in Section 2.3.2. The combinations of these available methods were tested exhaustively under the assumption that different users would need different learning pipelines to achieve their best results and that the f1 metric from in-session testing could be used as a predictor for valid models.

To explore how robust models trained on a single data set would be to new data obtained after subjects had a chance to relax and move around, models were trained on a 35/65 train/test split of the samples contained in Data1. As a baseline the trained model was validated in-session (IN) on the held out samples from Data1. The model was then used for inter-session (IS) validation on the samples from Data2. Confusion Matrices (CM) were generated for both IN and IS testing to compare accuracy and points of confusion. Two different methods for handling problems discussed in the introduction were explored. The FILT method uses an unsupervised outlier detector to identify actual transition points within the ‘Null’ repetitions. In this study a simple rule set was applied offline to correct class labels preceding and following the actual gesture transition portion of the ‘Null’ class. Samples flagged as outliers were dropped. Samples prior to first outlier in a ‘Null’ rep were relabeled as previous class, samples after last outlier were relabeled as next class; this was conducted to account for delay in beginning of ‘Null’ during which user was parsing next requested gesture, and end portions of ‘Null’ where user had finished transitioning to next gesture. A conventional classifier was then trained to recognize remaining gestures on Data1 and validated on Data2. In real time applications this could be envisioned as two separate stages of classification. In the first stage the outlier detector rejects samples representing transitions between gestures, and those samples not rejected are passed on to a model for classification. FILT is explained further in Section 2.3.4 of this manuscript. The Time Series Consolidation (TSC) method uses an algorithm designed for this paper to detect gestures which cannot be differentiated from each other meaningfully due to intrinsic limits in either the sensor system or users’ physical capabilities. TSC is explained Section 2.3.5 of this manuscript. For clarity methods not using the TSC method are denoted as SIS. The effect of each method was examined as an individual pipeline (FILTSIS, RAWTSC) and as a combined pipeline (FILTTSC). The results were then compared to the baseline results of intersession testing with no applied pipeline (RAWSIS). 

#### 2.3.2. Preprocessing (Feature Sets and Normalization)

Individual capacitive sensor samples were used to engineer a rate of change feature by subtracting the prior reading from the current reading as:

(2) $$Sensor_{delta}=Reading_{t}-Reading_{t-1}\;$$

The primary feature sets used were Cap (all capacitive strap readings), CapDelta (the sensor delta features), and Caps (all features in Cap and CapDelta). To test the effect of eliminating redundant and highly correlated features, each feature set was fed into the *featurewiz* library. This library that performs automatic feature selection via the SULOV algorithm and while the full details are outside the scope of this manuscript, they can be found in the associated reference [21]. The feature sets generated by *featureviz* were denoted OptimCap, OptimCaps, and OptimCapDelta. Feature engineering was applied to each dataset individually as it is a mathematical operation rather than a trained model.

While some algorithms such as Random Forest (RF) and K-Nearest Neighbor (KNN) work well with data that has variation in its range of values; most algorithms require that features be on the same scale to achieve their best performance. The two most common forms of data scaling are Normalization and Standardization.

In normalization, raw features are scaled to values between the range of (0,1) via the following formula:

(3) $$Feature_{normalized}=\frac{Feature-Feature_{MIN}}{Feature_{MAX}-Feature_{MIN}}\;$$

Standardization is a method of scaling that makes the assumption that all incoming data will fall into a Gaussian distribution and scales using the following function:

(4) $$Feature_{scaled}=\frac{Feature-\mu }{\sigma }\;$$

Both Normalization and Standardization make an implicit assumption of stationary and time invariant data, meaning that the minimum, maximum, mean, and standard deviation will remain fixed over time. As such, user motions and shifts in position could invalidate these assumptions and lead to more significant error than raw data. Raw data, data normalized via the MinMax scalar function in *scikit-learn*, and data standardized using the StandardScalar function in *scikit-learn* were provided as scaling options in the learning pipelines. To prevent data leakage between Data1 and Data2, models used for scaling were trained on a 35% split of Data1. The trained models were then used to scale the samples in Data1 and Data2.

#### 2.3.3. Unsupervised Transition Detection and Label Correction

Unsupervised models are commonly used for outlier detection in machine learning applications, and it was assumed that the motions representing the actual transition points could be treated as outliers relative to the normal data. Based on good preliminary results relative to other unsupervised models the Elliptical Envelope model was selected for outlier detection. The Elliptical Envelope model is an unsupervised classifier that uses covariance estimation on an assumed Gaussian distribution [25]. It attempts to group all the non-outlier data into an elliptical cluster, and assumes any data not fitting that cluster is an outlier. It was trained on a 35% random split of the ‘Null’ labeled samples from Data1 using a feature set composed of the CapDelta features discussed in Feature Sets.

The only hyper-parameter used in Elliptic Envelope is contamination, which is the assumed percentage of the data that is an outlier. This value cannot be known a priori as the time required for users to change gestures is not consistent. Unrequested motions on the user’s part may contribute to outliers, and only a small portion of the ‘Null’ samples are correctly labeled. To automate hyper-parameter selection, an initial contamination (contam_init_ = 0.25) was set. The corruption hyper-parameter was tuned by seeking a value that maximized the number of outliers found in the ‘Null’ class while minimizing the number of outliers found in other classes when validating on the held out portion of Data1.

The actual number of samples representing transitions and unrequested motions cannot be known a priori. Transitions between gestures should be found in the ‘Null’ class, and for are likely to make up a very small portion of that class. Conventional accuracy metrics are not meaningful as an accurate unsupervised outlier model will only label a very small portion of the ‘Null’ class as representing transitional motions and the unlabeled samples will be interpreted as many False Negatives. Using Precision instead of Accuracy avoids incorrectly penalizing samples in ‘Null’ not identified as outliers.

(5) $$Precision=\frac{True\;Postive}{True\;Postive+False\;Positive}\;$$

In Precision, the outliers which fall within ‘Null’ are True Positive, and those not falling in ‘Null’ are False Positives. Using Precision instead of Accuracy avoids incorrectly penalizing samples in the ‘Null’ not identified as outliers. The value of Precision closest to 1 was used to identify the best model settings as it indicated a maximum number of transition outliers being detected in ‘Null’ while minimal transition outliers were being placed incorrectly into other gesture categories.

To calculate the Accuracy of the FILT method, three assumptions were made: (i) Samples identified as outliers within ‘Null’ were True Positives, (ii) Samples not identified as outliers were True Negatives, (iii) Samples identified as outliers outside of ‘Null’ are False Positives. This allowed accuracy to be calculated as:

(6) $$ACC_{Stage1}=\frac{Outliers\;in\;Null+Non\;Outliers}{Total\;Number\;of\;Samples}\;$$

**Figure 3:**
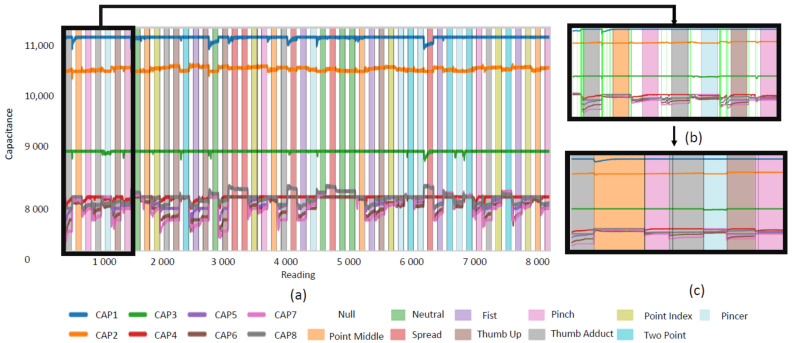
(**a**) Raw data from the sensors for a sample subject; (**b**) blow up of boxed section showing transitions detected by the unsupervised learner in lime green; (**c**) the same section of the data after dropping transitions and expanding class labels.

The outlier labels were used to remove and relabel samples as appropriate. In Figure 3b, the samples which were flagged as Null are shown in lime green. As can be seen, this leaves significant portions in the Null class that likely belong in neighboring classes. A rule was set to relabel samples prior to a flagged sample into the previous class and relabel samples after a transition into the next class. If multiple points were flagged in a Null class, the points between them were dropped. Figure 3c shows the results of applying these rules. The unsupervised model was applied to Data1 and Data2. The results of datasets on which the unsupervised model had been applied to filter out actual transitions correct labels are denoted as FILT.

#### 2.3.4. Supervised Learning Models

The quantity of data that could be generated in the time allotted to run tests on subjects was not adequate to build conventional deep learning models effectively. This limited the options available to classical classification models. Both K-Nearest Neighbor (KNN) and Support Vector Classification (SVC) are popular models in the context of gesture recognition. Under the assumption that the consolidation methods discussed in Section 2.3.5 might create significant class imbalances, the Balanced Random Forest (BRF) classifier from the *imblearn* library was also introduced as an option [26].

KNN works under the assumption that if the N nearest samples as selected by a distance metric fall into a given classification, then the current sample does as well. Both the number of neighbors and the distance metric are hyper parameters that can be tuned to optimize performance. It is noted by Ehsani et al. that often that the Manhattan distance outperforms traditional Euclidean distance measures [27]. It is also noted by both Ehsans et al., and Alfeilat et al. that the number of neighbors needed for optimal performance cannot be known a priori [27,28]. Based on preliminary results, Manhattan and Euclidian distance parameters were both given as options for distance metric, and options for number or neighbors included: 19, 21, 23, 25, 27, 29, or 31. The f1 score validated on Data1 was used as a metric to select the best set of hyper parameters for a given user.

SVC works by casting data to a higher dimensional representation and attempting to draw vectors between the classes maximizing the distance of separation between them. While SVC in its most basic form is used in linear models, it can use mathematical functions known as *kernels* to improve model performance on non-linear data [29]. SVC is very sensitive to unscaled data and generally does not generally achieve good performance on raw data [30]. For SVC, hyper parameter tuning was not explored in this work as there was not a single case in which it achieved anywhere close to the top accuracy on subject data.

BRF is a variation on the Random Forest algorithm which randomly under-samples more prevalent classes in order to reduce the effect of class imbalances [27]. Random Forests themselves are robust to unscaled data, and can often produce robust results on unscaled data, but are somewhat prone to overtraining on noise in the data. They work by creating ‘decision’ trees to classify the samples based on random selections of subsets of the features provided to them. Early results showed that best performance was achieved using 200 trees and leaving other settings at default.

#### 2.3.5. Inter-Session Trials

Initial feasibility testing pointed to a 35/65 train/test split as being a reasonable division of the samples in the Train set to avoid overtraining. During this initial feasibility testing it was observed that many subjects were inconsistent in forming gestures. Two forms of inter-session trials were explored in this work.

In inter-session trials, a 35/65 train/test split of the shuffled samples from Data1 containing an evenly balanced distribution of gestures was used to train a model; IN validation was performed against the held out samples from Data1 and IS using the samples in Data2.

To address the fact that subjects are not always consistent in production of gestures an alternate algorithm to train models is proposed. This algorithm which we shall call Time Series Consolidation (TSC) uses a variation on time series (TS) splits of the samples rather than a standard shuffle split. It averages the CM from each split and uses the averaged CM as a tool to find and merge classes of gestures likely to be confused with each other for a given subject. To make the potential user experience more intuitive, rules are set such that if one of the confused labels or ‘Fist’, or ‘Neutral’ that label is retained and the confused class is merged into ‘Neutral’ or ‘Fist’.

In TS splits, it is assumed that features comprising a sample may not be time invariant. To apply the TS split each gesture is divided into equal time ordered batches (*b_MAX_*) representing the discrete repetitions of the gesture performed by the user. For each batch (*b*) a model is trained and validated against the next batch (*b +* 1), and a confusion matrix (CM) is generated. Once all time steps have been tested, the CM are summed and divided by the number of batches used to obtain an arithmetic average:

(7) $${\mathrm{CM}}_{\mathrm{AVG}}=\frac{\mathrm{CM}}{b_{MAX}}$$

CM_AVG_ is then passed to a first Threshold Algorithm (T1) to consolidate regularly confused gestures as described in pseudocode in Table 1 as Threshold Algorithm 1 (T1). The CA takes a starting threshold (*thresh =* 0.99), a minimum threshold (*thresh_MIN_* = 0.7), the number of gestures we want the algorithm to find (*g_TARGET_* = 10) ($g_{TARGET}=10)$, and a minimum number of gestures (*g_MIN_* = 5) as inputs. It is initialized with the assumption that no gestures are recognized (*g_RECOG_* = 0). An iterative process is then performed on the data until the exit condition is reached and the values obtained for *thresh* and *g_RECOG_* are returned. The value for *g_RECOG_* can be either an integer value of gestures recognized or Fail if the pipeline fails to recognize any gestures.

If *g_RECOG_* ≠ *Fail* a Consolidation Algorithm (CA) is called using *thresh* and *g_RECOG_* as inputs to determine which classes should be merged by parsing through the CM_AVG_ matrix one line at a time. In this algorithm the rows (r) of the CM correspond to a given gesture label, and CM_rr_ indicates the accuracy for a given label. If the accuracy is below the threshold, then the label (g_r_) is put into a dictionary mapping it to the most commonly confused gesture label (g_j_) on that row of the CM. This mapping was applied relabel samples in both Data1 and Data2. The pseudocode for this algorithm is also in Table 1 as Consolidation Algorithm (CA).

This first prediction is further refined by generating a second confusion matrix (CM_2_) using a standard IS method on the relabeled Data1. This second threshold algorithm (T2) takes the threshold found in T1 as its starting threshold *thresh_START_*_2_ = *thresh* and a preset minimum number of classes (*g_TARG_* = 5). It adjusts the number of classes using the logic shown under pseudocode on Table 1 as Threshold Algorithm 2 (T2).

These new values for thresh and *g_RECOG_* are used as inputs to a second round of the (CA) and these mappings are applied to both for the pipelines using the TSC method. After merging classes in both the Data1 and Data2, the working model is trained on the Data1 and validated against Data2 to predict real world performance on the merged labels. In order to evaluate the effects of tailoring the grouped classes to individual subjects the accuracy of the RAWSIS is used as a baseline and compared to the accuracy of RAWTSC, FILTSIS, and FILTTSC methods in Results and Discussion.

#### 2.3.6. Metrics

In order to find the presumed best pipeline for each user, f1 was used as a metric to compare performance on various pipelines on Data1 and to select which one should be used on Data2. The f1 score is the harmonic mean of Precision and Recall and is a better predictor of performance on imbalanced multiclass problems than accuracy alone [7]. To generate an f1 score, first Precision and Recall scores are generated. Precision was discussed in Section 2.3.3. Recall tells us how likely a model is to capture all members of a class; it can be defined as $\frac{TP}{TP+FN}$. False Negative (FN) is the number of samples that should be in a class but are placed into a different class by the model. These two values for each class can be combined to generate an f1 score which gives a sense of how well a model is likely to generalize to real world performance.

(8) $$f1=\underset{i}{\sum }\frac{n_{i}}{N}\frac{\left(Precision_{i}*Recall_{i}\right)}{Precision_{i}+Recall_{i}}\;$$

In this equation, *N* is the total number of gesture classes, and *i* is the individual class. By summing the individual class performances and averaging, we can balance the performance across all classes present and account for class imbalances.

The confusion matrix used in Section 2.3.5 to calculate performance for TSC trials is a common tool used to analyze the performance of classification models. It consolidates accuracy, Type 1, and Type 2 error into a single table. Type 1 errors are analogous to FP and count the samples in a given class that the model places in the wrong class. Type 2 errors are analogous to FN and count the samples a model places in a given class that belong to a different class. The diagonal of a confusion matrix gives us individual class accuracies, and one can read across the rows to find Type 1 errors, and down the columns to find Type 2 errors.

(9) $$\mathrm{CM}=\left[\begin{array}{ccc} Acc_{1} & \ldots & Type\;2\;Error_{1,n}\\ \vdots & \ddots & \vdots \\ Type1\;Error_{n,1} & \ldots & Acc_{n} \end{array}\right]\;$$

For example, if we are interested in class 1 out of N classes, we would find the accuracy in the upper left corner (CM_11_), Row 1 would give us Type 1 error and how much occurred in each class. Column 1 would give us the Type 2 error and classes in which they occurred.

While we use the f1 value from Data1 to select models, it is less interesting from a use case standpoint than the number of classes retained after applying the TSC method and the accuracy with which those classes are predicted in the Data2 dataset. If too few classes are retained after merging, then the system is unlikely to be a useful interface. If the average accuracy is too low, then even if many gestures are retained they are unlikely to be useful.

Both of these metrics have the potential to be affected by the inclusion of the unsupervised filter layer discussed in Section 2.3.3. If the unsupervised layer is effective in removing the ‘Null’ class, the number of classes found in the FILT models needs to be adjusted to account for the fact that the ‘Null’ class was removed:

(10) $$Class_{adjust}=Class_{count}+1$$

This will also affect the accuracy. The class averaged accuracy is an arithmetic mean of the accuracy of each individual class. Because the filter stage removes the ‘Null’ class, it needs to be accounted for using the following formula:

(11) $$Acc_{adjust}=\left(\frac{Mean_{arithmetic}Number_{classes}+Acc_{stage1}}{Class_{adjust}}\right)$$

In these formula $Class_{count}$ is the number of classes found by the models trained on FILT data, and *Class_Adjust_* accounts for the removal of the ‘Null’ class. To further evaluate how effective the consolidation method is, *Class_Bad_* is introduced as a metric for classes achieving less than 80% accuracy on Data2 validation.

(12) $$Class_{Bad}=\overset{i}{\underset{i=1}{\sum }}[C_{ii}<0.8]$$

#### 2.3.7. Comparing Methods

**Figure 4:**
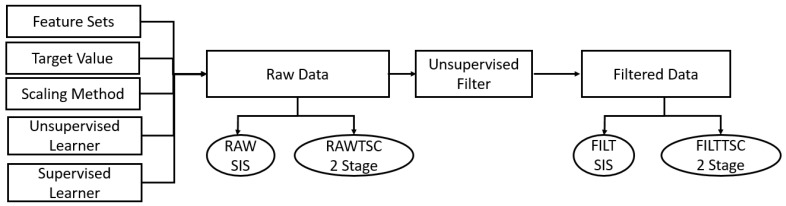
Machine Learning pipeline inputs and process flow leading to the output methods compared in this study.

There are four different methods of handling potentially mislabeled data and inconsistency in class production in this work. The baseline method (RAWSIS) neither filters nor consolidates. The results of applying TSC to the raw data are explored in (RAWTSC). The effects of filtering the data with an unsupervised algorithm are explored in (FILTSIS), and finally the effects of both filtering and consolidating data with TSC are explored in (FILTTSC). A conceptual diagram of where these methods split off from the general learning pipeline can be seen below in Figure 4.

### 2.4. Analysis

The number of subjects used for this study is not generally considered adequate to meet the assumptions of parametric statistical testing. In addition, as discussed in Results there is significant heteroskedasticity present in the data. This precludes the use of ANOVA to compare results. A common non-parametric stand in is the Kruskal-Wallis test which makes no assumptions regarding normalcy of the data and can achieve statistically meaningful results with as few as 5 samples and 2 or more groups [31,32]. The Kruskal-Wallis test only indicates whether there is a statistically significant difference between groups and if significant results are found. A Dunn test using a *p* correction term is applied post-hoc to determine which sets of data display significant differences [33]. Generally the Bonferroni correction is considered overly conservative, and most contemporary researchers use the Bonferroni-Holm correction instead to avoid Type 2 errors [34]. In this work the Bonferroni-Holm correction was applied to ensure that false importance was not attached to the results of different pipelines.

## 3. Results

The effect of using the unsupervised learner to find likely transition points and applying the rules discussed in Section 2.3.3 can be seen in Figure 3. On average, in-session the unsupervised learner had a Precision of 0.9912 (S.D. 0.0057) and an AccStage1 of 98.77% (S.D. 1.37%). In inter-session trails the Precision declined slightly to 0.9857 (S.D. 0.0148) and an AccStage1 of 93.8% (S.D. 4.46%).

The mean and standard deviation of accuracy and classes retained within the four methods considered were: RAWSIS (Accuracy = 42.47% ± 3.83%; Classes Retained = 11.00 ± 0.00; Class Bad = 9.38 ± 1.19), FILTSIS (Accuracy = 61.98% ± 9.17%; Classes Retained = 11.00 ± 0.00; Class Bad = 6.88 ± 1.89), RAWTSC (Accuracy= 93.03% ± 4.96%; Classes Retained = 5.29 ± 0.46; Class Bad = 0.5 ± 0.46), and FILTTSC (Accuracy = 87.32% ± 7.51%; Classes Retained = 6.57 ± 0.92; Class Bad = 1 ± 0.95). The results of the Kruskal–Wallis test showed a significant difference in both mean accuracy (*p* < 0.0001) and actual classes found (*p* < 0.0001). As seen in Table 2 the differences between SIS and TSC methods are statistically significant, but the differences between the two SIS methods and the two FILT methods do not reach statistical significance.

**Figure 5:**
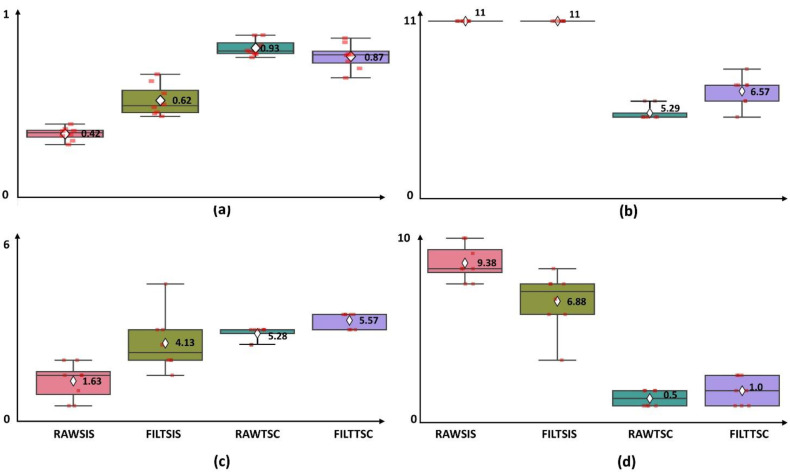
Box and scatter plots for (**a**) Accuracy vs. pipeline, (**b**) Number of classes retained vs. pipeline, (**c**) Number of classes that had more than 80% accuracy vs. pipeline, (**d**) Number of classes with less than 80% accuracy vs. pipeline. Note that in the FILT methods Acc Adjust and Class Adjust are used to account for the unsupervised layers removal of Null class.

Looking at the box and scatter plots in Figure 5 the unsupervised layer used in the FILT methods introduces significantly more heteroskedasticity than is seen in the raw data. TSC methods generally had better accuracy and less variance when trained on the raw data than when trained on filtered data.

The *p* values generated by running the Dunn test with Bonferroni-Holm correction in *scikit_posthocs* are shown on Table 2, with the values indicating statistical significance (*p* < 0.05) underlined. Both RAWTSC and FILTTSC show statistically significant differences in accuracy compared to the baseline of RAWSIS. The *p* value comparing the accuracy of RAWSIS vs. RAWTSC (*p =* 0.000016) is over an order of magnitude smaller than that of RAWSIS vs. FILTTSC (*p =* 0.000692); and when compared to the accuracy of FILTSIS only RAWTSC shows a statistically significant difference (*p =* 0.012379). It is tempting to assume that RAWTSC is the best pipeline overall, but the difference in accuracy between RAWTSC vs. FILTTSC does not reach the threshold of statistical significance (*p =* 0.37916). A similar trend applies to classes retained.

Table 3 contains a row for each machine learning pipeline along with a count of how many subjects each of the scaling methods, classification algorithms, and feature sets achieved the highest accuracy for. CapDelta and OptimCapDelta are not included on this table to save space as they did not produce the best results in any trials.

**Figure 6:**
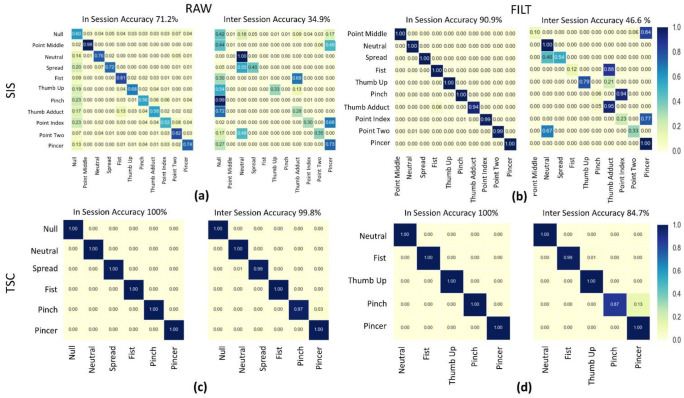
(**a**) RAWSIS results on both the Data1 (Left) and Data2 (Right) matrices; (**b**) The FILTSIS results on Data1 and Data2 show moderate improvements in accuracy, largely due to elimination of the Null class; (**c**) RAWTSC generally achieved the best accuracies; (**d**) FILTTSC has slightly lower accuracy overall even when the accuracy off the unsupervised stage in removing Null class is accounted for.

Figure 6 shows confusion matrices for all four data processing methods applied to Data1 and Data2 data from a sample subject. In order to compare the performance on in-set (IN) vs. inter-set (IS) testing the left hand side of each sub plot holds a confusion matrix for the results of applying a given pipeline of a train/test split taken from Data1. On the right hand side, a confusion matrix shows the results of applying the same pipeline but training on Data1 and validating on Data2. In all cases except for RAW the IN results for both 5-fold cross validation and shuffle split confusion matrices showed accuracy above 90%, but inter-set accuracy is highly variable across methods ranging from 34.9% to 99.8%.

Rules were set to preserve both the ‘Neutral’ and ‘Fist’ gestures because they were assumed to be the most intuitively different gestures for most users to make. Table 4 presents gestures retained by a sample subject after the RAWTSC method was applied. It can be seen that there was significant variability between subjects in regards to which gestures were retained. The most commonly retained gestures were the ‘Point Two’ (point with Index and Middle finger), and ‘Chuck Grip’ gestures. Otherwise there was significant variability in the gestures retained. Three of the subjects retained the ‘Point Middle’ gesture, but only two retained ‘Pinch’ or ‘Point Index’. Only one subject retained the ability to distinguish ‘Thumb Up’ and none of the subjects retained the ability to distinguish ‘Spread’ or ‘Thumb Adduct’ gestures.

The gestures retained by users with the FILTTSC method can be seen in Table 5. Because the purpose of the FILT method is to eliminate the transitional gestures within ‘Null’ and relabel the rest in their adjusting categories, all users are considered to have a detectable ‘Null’ category. However, it can be seen that six of eight users retained the ‘Point Index’ and ‘Chuck Grip’ gestures. While the ability to distinguish ‘Thumb Adduction’ was still limited to one of the subjects; ‘Thumb Up’ and ‘Spread’ could now be distinguished in three of the subjects.

## 4. Discussion

As mentioned in the introduction some assumptions were made when developing the learning pipelines. The underlying assumption of FILT is that the method used to assign ground truth while common in papers has a flaw baked into it that will lead to mislabeling of data. Correcting the labelling with a two-stage model should increase model accuracy both by improving the accuracy of the labels on samples used to train models, and insuring that the test data has a lower proportion of mislabeled samples. The underlying assumption of the TSC model is that users are not actually reliable or consistent in gesture production, and that by observing which gestures lose accuracy over multiple repetitions in one set, we can find those gestures likely to be mistaken for each other in different sessions. The TSC method identifies classes that are unlikely to be inconsistently produced by a given user over time leading to confusion with other similar classes. While an algorithm may over train on noise within a single session and achieve high accuracy on all classes, these classes are likely to have low accuracy in inter session trials. By identifying and merging those classes we can increase inter session accuracy and retain the more robustly recognized gestures for a given user. We also can control labels assigned to classes and report those labels back to the user resulting in a more understandable and intuitive class labels than would be produced by a purely unsupervised learning method. Finally, we looked at combining both FILT and TSC in a single pipeline to see if using them together would result in better performance than either one alone. For comparison purposes, all resulting accuracies were compared to the baseline performance of RAWSIS.

### 4.1. Unsupervised Layer

The development of the FILT method was motivated by the observation that even in-session accuracy had an unacceptably low value and the most common point of confusion was with the ‘Null’ class. As discussed in the introduction, the software labelling ground truth used in this and many other studies intrinsically creates mislabeled data [8,9,10,11,12,15]. When subjects are prompted to change gestures, the ground truth label is immediately switched to ‘Null’ and held for some period of time to allow transition (in this study 3 s). A typical class transition follows the following progression, (i) For some period in the beginning of the ‘Null’ class there is a lag while users remain in the previous gesture and parse the next requested gesture, (ii) a quick transition of the subjects’ hand position to the next requested gesture occurs, (iii) there is some period of time during which the user is now in the next requested class but the software has not updated labelling. In both phase i and phase iii the software provided ground truth labels will be erroneous. Examining the ‘Null’ class in Figure 4b shows that while these phases occur in each gesture transition the duration of these phases is not consistent.

In fact, as can be seen in Figure 6a, even for in-session tests the majority of inter class confusion involves the ‘Null’ class. It is a trivial (but tedious) matter in the lab to manually relabel data or discard data and commonly used in studies [8,9,10,11,12,15]. Doing so is not realistic for use outside a lab, and nor is using alternate technologies to validate ground truth as many home users will not possess alternate sensors. It makes sense intuitively that samples representing gesture transitions could be seen as outliers given that they represent only a small portion of the samples in ‘Null’. So, Outlier Detection was explored as a method to identify the samples within ‘Null’ representing gesture transitions.

As can be seen in Figure 5a, once the FILTSIS method is applied the mean accuracy achieved improved significantly over RAWSIS. It can also be seen in Table 3 that only one subject benefited from Caps feature set when the FILTSIS method was applied. This is likely because the rate of change on sensors should be near zero once a gesture is obtained and held. While filtering improved accuracy for the IS trials, it did not improve them enough that either of the SIS models would represent a useful gesture recognition interface given that the portion of gesture classes retained with accuracies above 80% was very low for both SIS methods.

When the FILTTSC method was used, more classes were preserved than by the FILTSIS method, but a lower mean accuracy was achieved. While this does not appear to be a statistically significant difference, the variance in both accuracy and number of classes preserved is higher for FILT methods in Figure 3a,b. Therefore, the outliers in the FILT methods have the effect of making the methods look more similar than they are. It is possible that this is a function of the rules used to relabel classes after detecting outliers, and that a more sophisticated ruleset could reduce the observed variability. It is also possible that a study with more subjects would reveal a statistically significant difference in performance between the FILT methods and RAWSIS. The FILTSIS method obtained an average inter-set accuracy of 61.98% while retaining 11 classes, which is somewhat lower than the 82.5% accuracy of Rossi et al. describe using their hybrid SVC-HMM model. However, their study only was performed on in-session data and limited itself to six gestures (including transitions) [13]. In a future study, it would be interesting to compare the results of the two methods directly on the same dataset.

### 4.2. Consolidation Algorithm

While the FILTSIS model had good accuracy when validated in-session, as seen in Figure 5, when inter-session validation was performed against samples from Data2 the models using it failed to generalize and displayed low accuracies. It was observed that for any given user there were sets of gestures commonly mistaken for each other, but that these confused gestures did not form a universal set across all users. It was observed that the same pattern of confusing gestures emerged when in-session tests were validated using inter-repetition train/test splits instead of shuffle splits. This led to the question of whether using multiple in-session, inter-repetition train test splits and averaging the results of the generated CM could uncover gestures that had poor inter-repetition consistency. As well, whether tailoring models to the individual user by merging those gestures most likely to be confused for that user would lead to more robust results for inter-session validation.

The TSC method is of particular interest because it fills a gap in terms of handling unreliable data and provides an ability to learn which gestures are valid for a given user. Current studies typically focus on a fixed number of gestures rather than tailoring the gestures in a model to a user’s capabilities [3]. Techniques that work with unreliable data typically use deep learning as a tool, but deep learning requires both a significant amount of data to learn from and may require significant computational power [4,7]. The field of concept drift can address nonstationary data, but as discussed in the introduction many contemporary studies on detecting gestures after sensor shift require fresh labeled data to learn from [20,21]. As discussed in Section 2.3.5, by leveraging the CM as a tool to consolidate confused gesture classes, the TSC method is able to learn likely areas of confusion without having fresh data injected into the model and can achieve good results using classical machine learning methods.

Typically, CMs are used as a visual tool to analyze where classification models break down, or to share results with human colleagues. By instead using it as an iterative tool to seek ‘clusters’ of errors, and the most common mistaken identities, the algorithm could preserve to some extent the labels initially provided by merging less distinct classes into the ones that were better recognized by the models. While unsupervised clustering can perform a similar function, it is less likely to do so in a way that preserves knowledge of class labels. The preservation of class labels should allow for a more intuitive user interface that is tailored to a specific user’s needs. Our method recognizes that the number and/or composition of classes that a sensor can recognize on a given user cannot be known a priori, nor can one know the threshold accuracy needed to segment out the subset of useful gestures or the best learning pipeline. The advantage of the TSC method is that it does not require this knowledge upfront. Instead, a larger set of gestures is requested from the user, and a starting *thresh* and *g_MIN_* = 5 are used to set a floor. The TSC method then seeks a subset of gestures that can be consistently recognized over several repetitions from the user. In the case of users who were consistent in their motions, these starting conditions have the potential to retain more than 5 gestures, but also ensure that the algorithm would preserve at least five gestures. This floor was set because it would be theoretically sufficient to allow the sensor system to perform basic mouse functions. While for healthy users this is merely a convenient function, for users possessing physical disabilities being able to tailor a gesture set used as input for their physical capabilities offers the potential to vastly improve their user experience.

The parameters of the study are somewhat comparable with Asfour et al.’s 2021 study. Similar to their study only a short break was used between sets. While the Asfour study used 16 gestures vs. the 11 used in this study, they achieved a lower accuracy of 86.4% ± 8.6% in-session and 78.5% ± 11.00% inter-session [11]. While they retained all gestures, they explicitly state that they only used static data from gesture classes and did not include a ‘Null’ or transitional class in their modeling. It seems likely doing so would result in losses of accuracy, but it would be of interest in the next stage of research to directly compare the results of applying their method to data collected on our subjects to the results obtained from the FILT and TSC methods. For all users, extrapolating the gestures likely to be performed consistently over time from a small dataset taken at the start of a user session rather than requiring multiple batches of fresh labeled data is a has the potential to improve the general user experience of wearable gesture recognition devices.

### 4.3. Combining TSC with Unsupervised Learning

All models had fairly good accuracy when validated using IN tests. However as seen in Figure 5, when exposed to new samples from Data2 the models using the RAWSIS and FILTSIS failed to generalize and displayed low accuracies. This could largely be attributed to the bias versus variance tradeoff. In other words, models learned the noise in the dataset rather than generalizable patterns.

The highest mean accuracy and lowest variability (Figure 5) were achieved using the RAWTSC method, which also had the least variability in the useable number of classes. While the RAWTSC method retained fewer classes after merging, the classes it retained had far higher IS accuracy. This is attributed to the fact that most subjects did not consistently perform all 11 of the gestures used in the study. In some cases, this can be attributed to the physical capabilities of the individual subject. For example, one of the subjects indicated that they suffered from arthritis, and another indicated that they had mild carpal tunnel. Other users indicated that they had difficulty making specific single-finger gestures, or that they experienced mild fatigue over the course of the experiment which could also lead to inconsistent production of gestures.

It was surprising that FILTTSC app”ared’to lead to lower mean accuracy and higher variance, but did not reach the criterion for statistical significance. It is possible that the variance itself was the reason for the lack of statistical significance as it can be seen that while six of the users had lower accuracies than the average values seen for RAWTSC, two of the users were high accuracy outliers. While the difference in accuracy does not meet the criterion for statistical significance, the variance may point to further work being needed in the rules-based algorithm that accompanies the FILT method. While the single unsupervised model could detect outliers of transitional gestures, it could not meaningfully detect incorrect gestures.

It was noted that for some subjects the accuracy for IN validation was at 100% for all gesture classes, but declined significantly for one or two gestures in IS validation. For almost all the subjects observed, there were instances in which the gesture they made was not the one prompted by the application. In order to test the robustness of the detection and consolidation algorithm in a more realistic use case, these mislabeled gestures were not removed. In cases where these mislabeled gestures were present in Data1 they would represent merging mislabeled data into a class; if present in Data2 they could account for some of the observed error. Training an unsupervised learner to recognize outliers in each gesture class could potentially do a better job of filtering out user error prior to relabeling and classification.

### 4.4. Scaling and Models

A glance at Table 3 shows that the majority of the time the best results were achieved using unscaled data. However, in the case of RAWTSC; scaling via Standardization had a slight edge on performance compared to other scaling methods. This is assumed to be due to two factors: (1) in many cases subjects made extra and unrequested motions (including adjusting their facemask in one notable instance) which could result in measured values with no relationship to the expected range of values during gestures, (2) the assumption that data is stationary is violated due to variations in position and how the subjects made gestures between datasets. If only small differences existed between classes, the adjustment of feature values that occurred when standardization and normalization were applied could be enough to blur the boundaries between classes. The only case in which better performance is achieved scaling via Standardization is in the RAWTSC method where Standardization is present twice as often as Normalization and only two subjects achieve their best performance with unscaled data. Because the TSC method acts to merge the classes most likely to be confused with each other, it is not surprising that scaling is able to improve results. It is however unexpected that TSC performs better on RAW data than the FILT data, and it is possible that using a more sophisticated ruleset is needed for the transitional data. The current ruleset simply labels all samples between the previous class and the outlier as being in the previous class, and all samples between and outlier and the next class as being in the next class. This could inadvertently capture samples that do not belong in any class.

Because the goal was to tailor the model to the user, no assumptions were made regarding which models would best fit a given user; instead, the f1 metric discussed earlier was generated on the results of IN validation and used to select the model that presumably would have the best IS performance. Across all the methods explored, the best performance comes from KNN (though the number of neighbors needs to be tailored to a given subject by exploring the f1 scores on Data1). The performance of BRF improves slightly when using the FILT and TSC methods. This is likely because filtering and consolidating data leads to imbalances in class composition and the imbalanced learning model can handle better than standard KNN. In all instances, SVC performs poorly, and never achieves the best accuracy for any user or method. This may also be tied to the underlying lack of stationary data.

Automatic feature reduction did not significantly improve results. In the RAW data, using the Caps feature set was often needed to achieve good results. This is possibly because it is easier to detect Null motions with the rate of change features. In filtered data sets the extra data encoded in the Caps feature set tends to not make models more effective as the Null motions have already been stripped by the unsupervised learning layer.

### 4.5. Gestures Retained

There was no universal set of gestures retained which points to the retained gestures being unique to a given subject rather than a set that could be created a priori and applied to all users. This is likely due to two factors. Firstly, different users likely had different physical capabilities in terms of which gestures they could continue to make distinctly over time. Secondly, the capacitive sensors measure the deformation of the forearm muscles as different gestures are made. While the volume of muscles remains constant, the actual flexed and relaxed portions of muscle will change the distance of the skin from the strap sensors at different cross sections along the arm. A subject’s forearm muscular development and specifically, the muscles that a subject engages for a particular gesture, are likely a function of the subject’s daily activities and hobbies. Finally, while there were variations in arm length, subcutaneous fat, and musculature of the users, the sensor sleeve itself was a ‘one size fits all’ configuration.

RAWSIS and FILTSIS preserved all gestures, but not at a high enough accuracy to be particularly useful. While the two TSC methods preserved five to seven classes per subject; there was significant variation in the classes preserved both by subject and by the method. For all subjects, rules settings ensured that ‘Null, ‘Neutral’, and ‘Fist’ would be preserved. The rejection of ‘Null’ by the unsupervised learner in FILTTSC ensured that it would be recognized as an outlier, and in the RAWTSC method, ‘Null was distinct enough for other gestures that the system preserved it. This is potentially useful in rejecting non-gesture motions. In general, when more fingers were involved in making a gesture, it was more likely to be retained as a unique gesture.

As seen in Table 4, ‘Pinch’ and ‘Point Two’ were identifiable in half the subjects and otherwise, there was significant variability in which gestures could be recognized for the RAWTSC method. A common trend in RAWTSC was that ‘Spread’ and ‘Thumb Adduct’ were not recognizable for any subject, and that ‘Thumb Up’ was only recognizable on one of the subjects. It is tempting to attribute this to the fact that the majority of the muscles used to spread the fingers and move the thumb are intrinsic to the hand. However, as can be seen in Table 5 a significant number of classes are recovered when the ‘Null’ class is eliminated by the unsupervised learner in the FILTTSC method.

The gesture ‘Point Index’ is retained for six subjects with reasonable accuracy. ‘Point Middle’ is retained for five subjects, albeit for two of the subjects it proves to be a very low accuracy class. The ‘Thumb Up’ and ‘Spread’ gestures can be recognized in three of the subjects and while ‘Thumb Up’ has good accuracy, ‘Spread’ never achieves an accuracy of > 60% for any of the subjects retaining it as a gesture. One of the users also retains the ‘Thumb Adduct’ gesture with high accuracy.

A possible reason for the challenge of recognizing the ‘Spread’ gesture and the various gestures involving the thumb is the location of the muscles used in these gestures. Muscles involved in spreading the fingers are located within the hand instead of the forearm and many of the muscles responsible for thumb use are also located in the hand. The muscles extrinsic to the hand which controls the thumb are relatively small muscles and are located beneath other larger muscles. It is likely that the difference between the gestures involving the thumb and other gesture classes is not large enough to be distinguished in the SIS methods and they only become clear after some classes are consolidated. There does not seem to be a distinct pattern for weight, arm length, or gender in regards to which subjects retained these gesture classes. It should be noted however that ‘Spread’ and ‘Point Middle’ are often the lowest accuracy classes and adding a rule to prevent them from being retained by the TSC method may improve the overall performance and usability of the system as a human-machine interface.

### 4.6. Limitations

This pilot study represents a first step towards validating both the FILT method of handling label inaccuracy within the ‘Null’ class and the TSC method of tailoring gestures to user capabilities. That said it is a small pilot study with only eight users. We used a small number of subjects and in order to ensure that we would not lose subjects to attrition, we collected both data sets in a single day. While several users self-reported carpal tunnel or arthritis as conditions that affected their hand mobility and comfort in making certain gestures, this is anecdotal rather than statistical information. A much larger number of subjects will be needed in future studies to determine if patterns emerge related to physiological and anatomical variations. To determine whether tailoring gestures to users represents a significant improvement in gesture recognition interfaces for disabled users, the next study should intentionally recruit from disabled populations as well.

In this study, an armrest was used to assist subjects in maintaining a consistent position of their wrist and arm throughout the data recording sessions. Many studies point to challenges in gesture recognition when multiple arm positions are added to the training and validation sets [10,15,35]. Without including both static and active changes in wrist and elbow position the robustness of models created in this work to changes in user position is unknown. Especially in the case of FILT methods, valid gestures may not be recognized or may even be rejected by the first-stage model. Several studies also indicate that challenges in inter-day tests emerge that due to changes in placement and positioning of sensors that may not be observed in single-day tests [10,15]. Further studies in which the subjects are instructed to maintain or make hand gesture poses while changing the wrist and elbow position will help determine how sensitive models are to changes in the subject position. Ideally, these data sessions should be recorded on multiple days to determine if the sensitivity to placement and body conditions between days is a confounding factor.

A metal hook sewn to an elastic strap was used in conjunction with an evenly spaced set of eyes to ensure that the straps would start with a consistent pressure on the subjects’ arm in the rest in a ‘Neutral’ starting position. This introduces the possibility that as subjects move away from the position the model was trained in, variations in pressure or position of the straps will be introduced and lead to model errors. One possible way to validate this would be to use pressure sensors. Pressures sensors have seen use in gesture recognition, but could also provide useful ground truth on changes in limb pressure against the capacitive straps used in this study [3,36]

The capacitive strap sensors used in this study only measure a change of capacitance caused by cross-sectional area change of muscles on the forearm as they contract or relax. This limitation likely prevents gestures dependent on the intrinsic muscles of the hand such as ‘Spread’ and ‘Thumb Adduct’ from being recognized effectively by this type of sensing system. While noise within a dataset may be falsely recognized as an indicator for the ‘Spread’ gesture, it does not generalize well outside of a dataset. The next set of experiments should either use a hybrid sensor approach to track gestures such as thumb motions and spreading of fingers which are controlled by muscles intrinsic to the hand.

## 5. Summary

In this study, we investigated methods to mitigate two confounding factors in gesture recognition (i) inaccurate ground truth for of samples representing transitions between gestures caused by software-based labeling, (ii) inconsistent production of gestures by users. We used capacitive strap gesture recognition sensors, and collected two data sets (Data1, Data2) containing five repetitions of ten gestures and a ‘Null’ category for gesture transitions from eight subjects. Data1 was used for training models and Data2 for validation in order to provide more realistic inter-session assessments of performance.

Two methods were developed to resolve these issues. FILT used an unsupervised learner to find outliers in the ‘Null’ class under the assumption these outliers represented gesture transitions. Samples flagged as outliers were dropped and labels of the remaining samples in the ‘Null’ class were reassigned to preceding or the following class as appropriate. The relabeled samples were then used to train and validate models trained using a conventional shuffle split. TSC created a series of inter-repetition models and averaged their confusion matrices to find the gesture classes unlikely to be consistently produced by a given user. The labels for these classes were then merged and a conventional learning model was trained. In both cases, models were trained on Data1 and validated on Data2.

Four pipelines were tested consisting of a mixture of these methods. RAWSIS used unmodified samples, and RAWFILT applied only the FILT method. RAWTSC applied only the TSC method, and FILTTSC applied the FILT method and then the TSC method. RAWSIS had the lowest average performance. It achieved an average accuracy of 91.16% ± 6.70% for in-session validation on Data1, and 61.98% ± 9.17% for intersession validation. The best performance was achieved by RAWTSC with an average intersession accuracy of 93.03% ± 4.96%.

## 6. Conclusions

The focus of this study was automatically correcting inaccurate ground truth labels for transitional motions and tailoring the number of gestures our sensor system included in its classification model to the physical capabilities of the user and sensor system with the goal of creating a more robust and intuitive human-machine interface. Methods improving the intersession accuracy of models should presumably allow longer use sessions with less need for the user to retrain devices. The FILT methods as implemented in this study appeared to preserve more classes compared to RAWTSC and provide modest improvements in accuracy as compared to the baseline of RAWSIS, but was not reach a threshold of statistical significance in either case. This may have been caused by the ruleset used to relabel classes around points of transitional motion. The accuracy of the RAWTSC method on the other hand produced significant improvements in accuracy as compared to both RAWSIS and FILTSIS. It is possible that fine-tuning the values of *classMin* and *classMax* could lead to optimal results in terms of maximizing both accuracy and number of classes retained by RAWTSC and this should be explored in future studies.

The TSC method allows for the number of classes that are modeled for a given user to be tailored to the physical capabilities of that user. This is a potentially important advance in that it allows for a more robust model to be developed which is tailored to the user; in specific it is tailored to the gestures they can make consistently and repeatedly over time, which allows for far better IS accuracy than conventional methods. It also has the potential to generate a model which is able to automatically recognize the subset of gestures that are likely to retain accuracy over time as a user fatigues and which are less likely to be affected by a given user’s individual disabilities. Further studies with groups of subjects including disabled users could produce assistive devices that adapt to the needs of a given user.

## Figures and Tables

**Table 1:** Algorithms used to assign thresholds and consolidate classes.

**Algorithm 1.** Threshold Algorithm (T1)	**Algorithm 2.** Consolidation Algorithm (CA)
1: **Set** Run = True	1: **Set** $g_{map}=\{\}$
2: **Set** $thresh=thresh_{START}$	2: **For** r in Rows:
3: **Set** $g_{MAX}=g_{TARGET}$	3: **If** $CM_{rr}<thresh$
4: **While** Run	4: **Set** $g_{j}=(\max C_{r,:})where\;j\neq r$
5: **Set** $g_{\mathrm{recog}}\;=\overset{i}{\underset{i=1}{\sum }}[CM_{avg,ii}\geq thresh]$	5: **If** $g_{j}not\;in\;g_{map}$
6: **If** $g_{RECOG}<g_{TARGET}$	6: **Set** $g_{map}\left(g_{r}\right)=g_{j}$
7: **If** $thresh>thresh_{MIN}$	7: **Else**
8: **Set** thresh=thresh−0.005	8: **Set** $g_{map}\left(g_{r}\right)=g_{map}\left(g_{j}\right)$
9: **Else**	9: **Return** g_ma_
10: **If** $g_{TARGET}>G_{MIN}$	**Algorithm 3.** Threshold Algorithm 2 (T2)
11: **Set** $thresh=thresh_{START}$	1: **Set** Run=True
12: **Set** $g_{TARGET}=g_{TARGET}-1$	2: **Set** $thresh=thresh_{START2}$
13: **Else**	3: **While** Run
14: **Set** $g_{RECOG}=Fail$	4: **Set** $g_{RECOG2}=\overset{i}{\underset{i=1}{\sum }}[CM_{2,ii}\geq thresh]$
15: **Set** Run=False	5: **If** $g_{RECOG2}>g_{TARG2}$:
16: **Else**	6: **If** thresh < 1:
17: **Set** Run=False	7: **Set** thresh=thresh+0.001
18: **Return** $g_{RECOG},\;thresh$	8: **Else**
	9: **Set** Run = False
	10: **Else**:
	11: **Set** Run = False
	12: **Return** $g_{RECOG2},thresh_{2}$

**Table 2:** Post Hoc Dunn Tests for Accuracy and Class Retention.

Adjusted Accuracy					Number of Classes Retained				
	RAW SIS	FILT SIS	RAW TSC	FILT TSC		RAW SIS	FILT SIS	RAW TSC	FILT TSC
RAWSIS	1.000000	0.166456	0.000016	0.000692	RAWSIS	1.000000	1.000000	0.000192	0.005821
FILTSIS	0.166456	1.000000	0.012379	0.112933	FILTSIS	1.000000	1.000000	0.000192	0.005821
RAWTSC	0.000016	0.012379	1.000000	0. 37916	RAWTSC	0.000192	0.000192	1.000000	0.659001
FILTTSC	0.000692	0.112933	0. 37916	1.000000	FILTTSC	0.005821	0.005821	0.659001	1.000000

**Table 3:** Scaling Methods, Classification Models, Features Sets as a function of Method.

	Scaling			Algorithm			Feature Set			
Method	None	MinMax	Standard	KNN	BRF	SVC	Cap	Caps	OtimCap	OptimCaps
RAWSIS	5	3	0	7	1	0	4	3	1	0
FILTSIS	5	3	0	6	2	0	5	1	1	1
RAWTSC	2	2	4	5	3	0	4	2	1	1
FILTTSC	6	1	1	6	2	0	6	1	1	0

**Table 4:** RAWTSC Gestures Retained and Accuracy.

User	Null	Point Middle	Neutral	Spread	Fist	Thumb Up	Pinch	Adduct Thumb	Point Index	Point Two	Chuck Grip
SUBJ20995	1	0.7	1		1						1
SUBJ49764	1		0.89		0.99	0.91				1	
SUBJ82743	1	1	1		1						1
SUBJ6420	1		0.88		1				1	1	
SUBJ54044	1		1		1		0.85				1
SUBJ68042	1		0.65		1					0.85	1
SUBJ77033	1		0.88		0.98				0.59	0.6	1
SUBJ82707	1	0.3	1		1		1				0.83
Count	8	3	8	0	8	1	2	0	2	4	6

**Table 5:** FILTTSC Gestures Retained.

User	Null	Point Middle	Neutral	Spread	Fist	Thumb Up	Pinch	Adduct Thumb	Point Index	Point Two	Chuck Grip
SUBJ20995	1	0.87	1		1		0.47	1			1
SUBJ49764	1		0.68	0	0.99	0.71			1		1
SUBJ82743	1	1	1		1				1		0.79
SUBJ6420	1	1	0.93		1				1	1	
SUBJ54044	1	0.14	1	0.55	0.99	0.98	0.93				1
SUBJ68042	1		0.63		0.96				0.90	0.89	0.96
SUBJ77033	1		1	0.59	0.92				0.98	0.51	
SUBJ82707	1	0	1		0.8	1			0.77		0.71
Total	8	5	8	3	8	3	2	1	6	3	6

## Data Availability

The data from this experiment has not been published online due to IRB requirements. A small dataset from the author can be made available on request.
